# Value of Foxp3 expressing T-regulatory cells in renal tissue in lupus nephritis; an immunohistochemical study

**DOI:** 10.15171/jnp.2016.19

**Published:** 2016-07-02

**Authors:** Marwa M Shakweer, Maha Behairy, Nadia G Elhefnawy, Tamer W Elsaid

**Affiliations:** ^1^Pathology Department, Faculty of Medicine, Ain Shams University, Cairo, Egypt; ^2^Internal Medicine and Nephrology Department, Faculty of Medicine, Ain Shams University, Cairo, Egypt

**Keywords:** Foxp3, Activity index, Chronicity index, Proteinuria, Lupus nephritis, T regulatory cells

## Abstract

**Background:**

Forkhead box P3 (Foxp3) functions as a master regulator in the development and function of T-regulatory (Treg) cells. Recent studies have shown that autoimmune diseases including systemic lupus erythematosus (SLE) are associated with an imbalance with the Treg cells and T helper (Th) subtypes.

**Objectives:**

To evaluate immunohistochemical expression of Foxp3 positive Treg cells in lupus nephritis (LN) and analyze its association with clinicopathologic parameters.

**Materials and Methods:**

Renal biopsy specimens of 50 patients with LN were studied. Specimens were divided into; group A; 25 LN cases without proliferative activity (Class II and V) and group B: 25 cases with proliferative activity (Class III and IV). Immunohistochemical staining for anti-human Foxp3 antibody and grading from grade 0 to grade 3 was done.

**Results:**

Foxp3 expression in group A was (grade 0 in 14 [56.0%], grade +1 in 11 [44.0 %]) in comparison to group B (grade +1 in 6 [24.0%], grade +2 in 11 [44.0%] and grade +3 in 8 [32.0%]) (*P* < 0.001). Foxp3 expression was significantly correlated to National Institutes of Health (NIH) activity and chronicity indices (*P* < 0.05), as well as serum creatinine (*P* < 0.01) in both groups A and B and there was a highly significant correlation with proteinuria (*P* < 0.01) in group B with proliferative LN.

**Conclusions:**

Immunohistochemical Foxp3 expression in renal tissue was higher in proliferative versus non-proliferative LN and is associated with activity and severity of LN. Further studies are needed to determine its prognostic value in LN.

**Implication for health policy/practice/research/medical education:**

T regulatory (Treg) cells are an important subset of T cells with important immunomodulatory effects. Foxp 3 is a key transcription factor of Treg cells. Its immunohistochemical expression has been shown to correlate with a number of clinical and histological parameters in a number of immune-mediated diseases. It is important to analyze the role of these cells in lupus nephritis, which is one of the common immune-mediated disorders. This study shows that this marker is highly expressed in proliferative lupus nephritis and correlated with NIH activity and chronicity indices indicating that Treg cells are implicated in disease progression.

## 1. Background

Lupus nephritis (LN) is the most important complication of systemic lupus erythematosus (SLE), which is responsible for SLE-related mortality and morbidity. LN occurs in up to 50% of patients with SLE (1). In LN, the deposition of immune complexes plays a leading role in the initiation of the disease. Subsequently, the deposited immune complexes and dendritic cells trigger the release of type I interferon and induce activation of infiltrating T cells, and this leads to intensification of T cell subgroups including the CD4+ T helper (Th) subtypes. Furthermore, CD4+ T cells are stimulated via the presentation of the putative antigens presented by dendritic cells or macrophages in the context of class II major histocompatibility complex (2,3). The Th cells have certain subtypes. Th1 and Th17 classically represent the inflammatory Th cells whereas the regulatory T cells (Treg) have a regulatory and suppressive role. Recent studies have detected that autoimmune diseases, including SLE, are associated with an imbalance with the Treg population and the inflammatory Th subtypes in the system. IFN-γ is the main cytokine product of the Th1 cells whereas IL-17 is the major cytokine of Th17 cells (4). Treg cells play a role in the prevention of uncontrolled immune responses against pathogens or allergens, guarantee stability of the normal microflora and prevent the escape of tumors from immune surveillance. Treg cells are also implicated in the development of cancer, autoimmunity, allergy and asthma (5-7).

Forkhead box P3 (Foxp3) protein is a transcription factor of the forkhead family, which is required for the development and function of most thymus-derived, naturally occurring Treg cells (8). This molecule also plays a crucial role in the regulation of immune responses mediated by peripheral T cells, preventing autoimmunity and permitting the maintenance of Treg cells (9). Foxp3 is the most specific marker of Treg cells currently available (10-12). Few studies investigating the presence and function of Foxp3 expressing Treg cells in renal tissue in LN are done (13,14).

## 2. Objectives

The objective of this investigation was to evaluate immunohistochemical expression of Foxp3 expressing Treg cells in LN and analyze its association with various clinical and histopathological parameters.

## 3. Materials and Methods

### 3.1. Study population

This study included 50 renal biopsy specimens that were diagnosed as LN at the pathology laboratory, and electron microscopy (EM) laboratory of Ain-Shams University hospitals during the period of 2012 to 2014. Data extraction sheet was designed to collect data retrospectively from records as personal data, medical history and laboratory results including serum creatinine and proteinuria by protein dipstick grading with approximate daily amount classified into grade I: 1+ dipstick (0.15–1.0g/24h), grade II: 2+ or 3+ dipstick (>1.0–3.5g/24h), grade III: 4+ on dipstick (>3.5g/24h) (15).

Cases with adequate paraffin tissue blocks for immunostaining were enrolled in the study.

All renal biopsy specimens were graded according to the 2003 International Society of Nephrology/Renal Pathology Society (ISN/RPS) classification system (16) and National Institutes of Health (NIH) activity and chronicity indices (17,18). The renal biopsy specimens were divided into:

Group A: LN cases without proliferative activity (Classes II and V)
Group B: LN cases with proliferative activity (Classes III and IV)

The renal specimens were re-evaluated by light microscopy (LM) and immunohistochemical staining using anti-human Foxp3 polyclonal antibody.

### 3.2. Light microscopy

Formalin-fixed, paraffin-embedded tissues from all cases were retrieved from the archive of the pathology laboratory. Slides were re-evaluated by two pathologists using LM (Olympus BX31) according to the histopathological guidelines for the evaluation and scoring of LN (16-18). The pathologists were blinded to the clinical parameters of the patients.

### 3.3. Immunohistochemical staining method

Tissue sections were deparaffinized, rehydrated, and the endogenous peroxidase activity was quenched by 10-minute incubation in 3% hydrogen peroxide in methanol. After antigen retrieval and protein block, the primary antibody (Rabbit anti-human Foxp3 polyclonal antibody; catalog number: E18491, spring bioscience) was supplied as 7.0 mL prediluted immunogen affinity purified rabbit polyclonal antibody in TBS/1% PSA buffer pH 7.6 with less than 0.1% sodium azide. The secondary antibody (supersensitive immunodetection system (Biogenex, catalog No: AD000-SL) was then applied followed by peroxidase labeled streptavidin. Slides were incubated for 10 minutes with substrate chromogen (DAB) mixture. Finally, the slides were counterstained and mounted with Canada balsam. Tissue sections from tonsils were used as positive control. Positive control slides were included in each run.

### 3.4. Semi-quantitative evaluation of immunostaining

Cells revealing clear lymphocyte morphology with distinct nuclear staining for Foxp3 but unstained cytoplasm (interpreted as Treg cells) were semi-quantified according to Yazici et al, (13) as follows:

Grade 0 was defined as the complete absence or weak staining in <1% of the cells; grade 1 was staining in 1%–10% of the cells; grade 2 was positive staining in 11%–50% of the cells; and grade 3 was positive staining in >50% of the cells. Glomerular and tubular staining with Foxp3 was not graded.

### 3.5. Ethical issues

The research followed the tenets of the Declaration of Helsinki. The work is a retrospective study which included only paraffin blocks.The research was approved by the Ethics Committee of Faculty of Medicine of Ain Shams Uiversity.

### 3.6. Statistical methods

Data was revised for its completeness and consistency. Double data entry on SPSS program version 16 was done. Quantitative data were summarized by mean along with standard deviation while qualitative data were summarized by frequencies and percentages. Analysis of variance, chi-square test and Spearman’s correlation coefficient test were used the analysis of significant differences or associations. A “*P* value” of less than 0.05 was considered statistically significant.

## 4. Results

**Figure 1:**
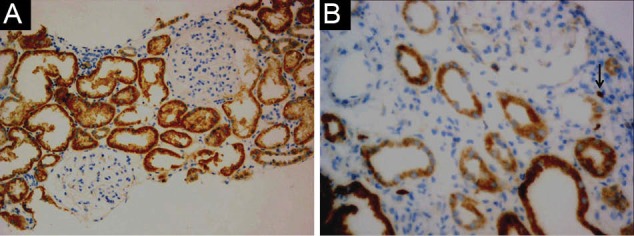
Class II lupus nephritis (group A) showing nuclear Foxp3 expression in <0%-1% of cells (arrow) (score 0). The renal tubules show nonspecific cytoplasmic staining with negative nuclear staining. Renal glomeruli show negative immunostaining (Original magnification: 1Ax200, 1B×400).

**Figure 2:**
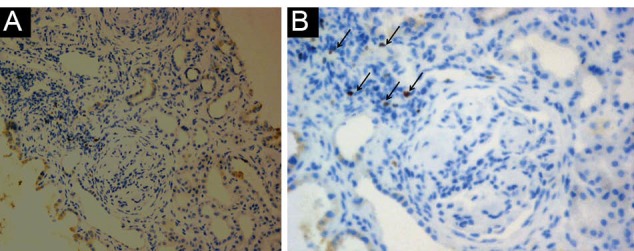
Class III lupus nephritis (group B) showing nuclear Foxp3 expression in <1%-10% of cells (arrows) (grade 1) (Original magnification: 2A×200, 2B×400).

**Figure 3:**
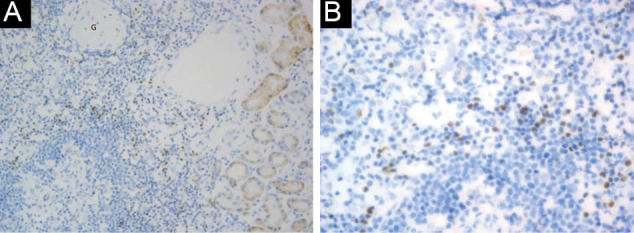
Class IV lupus nephritis (group B) showing a globally sclerotic glomerulus (G) with nuclear Foxp3 expression in <11%-50% of cells (grade 2). (Original magnification: 3A×200, 3B×400).

Fifty renal biopsy specimens with LN were re-evaluated and divided into two groups. Group A included 25 cases with non-proliferative LN (19 cases of class II, and 6 cases of class V). Among these, 24 (96%) were females and one (4%) was male, with mean age of 24.6 ± 12.7 years. Group B included 25 cases with proliferative LN (class III, IV), where 18 (72%) were females and 7 (28 %) males, with mean age of 29.3 ± 13.9 years. The patients’ characteristics, laboratory results and NIH activity and chronicity indices of LN of both studied groups are described in Table 1. Group B showed significantly higher mean serum creatinine and higher mean activity and chronicity indices compared to group A (*P*<0.001). Immunohistochemical expression of Foxp3 among studied groups is shown in Table 2 and Figures 1, 2 and 3. Group B showed significantly higher percentage of Foxp3 immunostaining with grade +2 and grade +3 in comparison to group A (*P*<0.001), while group A showed higher percentage of negative Foxp3 staining. Immuohistochemical expression of Foxp3 was significantly correlated to activity index and chronicity index in group A (*P*<0.05) and in group B (*P*<0.001). There was significant association between Foxp3 expression and presence of wire loops (χ^2^=7.26, *P*=0.005), fibrinoid necrosis (χ^2^=6.21, *P*=0.029), cellular crescents (χ^2^=6.57, *P*=0.023) and interstitial inflammation (χ^2^=4.48, *P*=0.032) by Fisher’s exact test in group B as parameters of activity of LN.

**Table 1:** Comparison between studied groups regarding baseline patient’s characteristics and laboratories

	**Group A (n=25)** **Mean ± SD**	**Group B (n=25) Mean ± SD**	***t***	**P**
Age (year)	24.6 ± 12.7	29.3 ± 13.9	1.2	0.2
S. Creatinine (mg/dL)	1.14 ± 0.2	1.96 ± 0.6	5.5	0.000^**^
Activity index	2.8 ± 1.4	9.8 ± 4.3	7.6	0.000 ^**^
Chronicity index	0.6 ± 0.9	2.9 ± 2.1	4.9	0.000^**^
	**No. (%)**	**No. (%)**	**χ** ^2^	**P**
Sex			5.3	0.02^*^
Female	24 (96)	18 (72)		
Male	1 (4)	7 (28)		
Proteinuria			8.0	0.04^*^
0	10 (40)	2 (8)		
+1	8 (32)	16 (64)		
+2	5 (20)	5 (20)		
+3	2 (8)	2 (8)		

^**^Significant at 0.01 level; ^*^Significant at 0.05 level.

**Table 2:** Comparison between the two studied groups as regards the grade of Foxp3

**Foxp3**	**0** **No. (%)**	**+1** **No. (%)**	**+2** **No. (%)**	**+3** **No. (%)**	**χ** ^2^	**P**
Group A (n = 25)	14 (56.0)	11 (44.0)	0 (0)	0 (0)	34.4	0.000^**^
Group B (n = 25)		6 (24.0)	11 (44.0)	8 (32.0)		

^**^Significant at 0.01 level

There was highly significant positive correlation between Foxp3 expression and serum creatinine in both non-proliferative (group A) and proliferative LN (group B) (*P*<0.001) and proteinuria in group B (*P*<0.01) but not in group A. There was no correlation between age or sex and Foxp3 expression in both groups (Tables 3 and 4).

**Table 3:** Correlation between Foxp3 and studied parameters in group A

	**r**	**P**
Age (year)	-0.129	0.5
S. Creatinine (mg/dl)	0.708	0.000^**^
Proteinuria	0.331	0.1
Activity index	0.425	0.03^*^
Chronicity index	0.490	0.01^*^
Sex^#^	χ^2^* =* 1.0	0.4

Spearman’s test: ** Significant at 0.01 level, *Significant at 0.05 level; ^#^Chi-square test.

**Table 4:** Correlation between Foxp3 and studied parameters in group B

	**r**	**P**
Age (year)	-0.150	0.4
S. Creatinine (mg/dl)	0.755	0.000^**^
Proteinuria	0.633	0.001^**^
Activity index	0.896	0.000^**^
Chronicity index	0.529	0.007^**^
Sex^#^	χ^2^* =* 3.9	0.1

Spearman’s test: ^**^Significant at 0.01 level, ^*^Significant at 0.05 level; ^#^Chi-square test.

## 5. Discussion

Foxp3+ Treg cells have been known as a major regulator of immune homeostasis through their immunosuppressive function. Th17 lineage is a CD4+ T cell subset that exerts its function by secreting pro-inflammatory cytokines and protecting host against microbial infections. The altered ratio between Foxp3+ Treg cells and Th17 cells plays an important role in the pathogenesis of immune-related diseases (19). Recent studies of Foxp3 expression and function provided critical novel insights into biology of Treg cells and into the cellular mechanisms of the immune homeostasis (20).

The majority of studies have investigated the role of Foxp3+ Treg cells in peripheral blood (21-23), with very few studies on immunohistochemical staining of these cells (13).

In the present study, we confirmed that Foxp3 expression level in active proliferative LN (group B) was increased with a highly statistically significant difference in comparison to LN with minimal histological activity (group A). These results are in agreement with Abbass et al, (24) who found that Foxp3 gene expression is higher in patients with active lupus compared to those with inactive lupus. They were concerned in their study with the clinical activity including SLAIDI score, while we were concerned with histological NIH activity index. We assume that the higher expression of Foxp3 in active LN (group B) indicates an increase in the number of Treg cells in an attempt to control the exacerbated immune response since Treg cells play a central role in inducing and maintaining immunologic tolerance and in suppressing immune responses. Shen et al (25) have given this explanation when they found a significantly higher expression of Foxp3 in lichen planus compared to control group. The increased Foxp3 in active LN can be also explained by recent mice and human studies that have demonstrated that Treg cells can be reprogrammed into a novel population, IL-17+Foxp3+T cells, phenotypically and functionally resembling Th17 cells under the complicated cytokine stimulation (19). Yazici et al (13) evaluated immunohistochemical expression of Foxp3+ Treg cells in renal tissue of LN and did not show in their study a significant decrease of Foxp3 expression with increasing disease severity or proliferative classes in SLE. They explained their results by suggesting that Th cells have plasticity and the Treg cells can convert to Th17 cells under inflammatory milieu. This plasticity of Treg cells may explain the lack of significant differences among proliferative and non-proliferative groups.

On the other hand, other studies reported inverse correlation between Treg cells in peripheral blood and clinical disease activity, where this inverse relationship was not proved by immunohistochemical study (26,27). This discrepancy in results between different studies was explained by Bonelli et al, (28) who defined a new subset of Treg cells, CD4+CD25−Foxp3+ T cells, that were increased with high disease activity, while CD4+CD25+Foxp3+ Tcells were inversely related to activity (26,27). Further immunohistochemical studies comparing the two Foxp3 Treg cells subsets on the histopathological level are recommended.

In our study, there is a highly significant positive correlation between proteinuria and immunohistochemical expression of Foxp3 in group B versus no correlation in group A. Bonelli et al (28) detected CD4+CD25−Foxp3+ T cells in the urine sediment of a patient with LN. Moreover they observed a significant correlation between proportions of CD4+CD25−Foxp3+ T cells and the extent of proteinuria in patients with renal involvement. They observed a decrease in proportions of CD4+CD25−Foxp3+ T cells after the initiation of therapy with cyclophosphamide. This was paralleled by a decline in the extent of proteinuria. Similar to these results, Wang et al (29) proved that urinary Foxp3 mRNA is markedly up-regulated in patients with active LN, and the level of expression is closely associated with the clinical and histological disease activity. They detected higher level of urinary Foxp3 mRNA in patients with no response to therapy than those with partial response or complete response. These data highlight the role of medications targeted for Treg cells for amelioration of inflammatory process in active LN. In this study we did not evaluate the grade of immunohistochemical expression of Foxp3+ Treg cells after immunosuppressive therapy as it was a retrospective study. Further studies with patient follow-up are recommended to detect the effect of medications on Treg cells in renal tissue.

Wang et al (29) recommended the use of Foxp3 mRNA in urine sediment as a non-invasive biomarker for assessing the severity. We also recommend the use of immunohistochemical expression of Foxp3 as a predictor of severity as there was significant positive correlation with serum creatinine, and NIH activity and chronicity indices in both groups in the current study.

## 6. Conclusions

Foxp3 expressing Treg cells in renal tissue were higher in proliferative versus non-proliferative LN and are associated with activity and severity of LN. Further studies are needed to determine prognostic value of Foxp3 expression in LN.

## Limitations of the study

Evaluation of Foxp3 immunohistochemical expression before and after receiving the treatment of active LN was a limitation of our investigation.

## Authors’ contribution

All authors contributed to the design of the research. MMS, MB, NGE, and TWE shared in analysis and interpretation of data. All authors drafted the first version. MMS, MB, NGE, and TWE edited the first draft. All authors reviewed and commented on final draft.

## Conflicts of interest

The authors declare no conflict of interests.

## Funding/Support

None.
